# *Clostridium difficile* flagellin FliC: Evaluation as adjuvant and use in a mucosal vaccine against *Clostridium difficile*

**DOI:** 10.1371/journal.pone.0187212

**Published:** 2017-11-27

**Authors:** Jean-François Bruxelle, Assaf Mizrahi, Sandra Hoÿs, Anne Collignon, Claire Janoir, Séverine Péchiné

**Affiliations:** 1 EA4043 Unité Bactéries Pathogènes et Santé (UBaPS), Univ. Paris-Sud, Université Paris-Saclay, Châtenay-Malabry Cedex, France; 2 Service de Microbiologie Clinique, Groupe Hospitalier Paris Saint Joseph, Paris, France; Instituto Butantan, BRAZIL

## Abstract

The immunogenicity of bacterial flagellin has been reported in different studies. By its close interaction with the immune system, the flagellin represents an interesting adjuvant and vaccine candidate. *Salmonella* Typhimurium flagellin has already been tested as adjuvant to stimulate mucosal immunity. Here, we assessed the ability of *Clostridium difficile* flagellin FliC to act as a mucosal adjuvant, first combined with ovalbumin as antigen and second with a *C*. *difficile* surface protein, the precursor of the S-layer proteins SlpA. Using ovalbumin as antigen, we compared the gut mucosal adjuvanticity of FliC to *Salmonella* Typhimurium flagellin and cholera toxin. Two routes of immunization were tested in a mouse model: intra-rectal and intra-peritoneal, following which, gut mucosal and systemic antibody responses against ovalbumin (Immunoglobulins G and Immunoglobulins A) were analyzed by Enzyme-Linked Immuno Assay in intestinal contents and in sera. In addition, ovalbumin-specific immunoglobulin producing cells were detected in the intestinal lamina propria by Enzyme-Linked Immunospot. Results showed that FliC as adjuvant for immunization targeting ovalbumin was able to stimulate a gut mucosal and systemic antibody response independently of the immunization route. In order to develop a mucosal vaccine to prevent *C*. *difficile* intestinal colonization, we assessed in a mouse model the efficacy of FliC as adjuvant compared with cholera toxin co-administrated with the *C*. *difficile* S-layer precursor SlpA as antigen. After challenge, a significant decrease of *C*. *difficile* intestinal colonization was observed in immunized groups compared to the control group. Our results showed that *C*. *difficile* FliC could be used as adjuvant in mucosal vaccination strategy against *C*. *difficile* infections.

## Introduction

*Clostridium difficile* is an anaerobic spore-forming Gram positive bacterium, commonly found in the environment and frequently isolated in hospitals. *C*. *difficile* is an enteropathogen responsible for diarrhea and pseudomembranous colitis associated to gut microbiota dysbiosis frequently consecutive to antibiotic therapy in the elderly [1].

European guidelines for treatment of *C*. *difficile* infection (CDI) consist of the discontinuation of CDI-inducing antibiotherapy and administration of specific antibiotics depending on the severity of the disease [2]. However, approximately 20% of treated patients experience recurrences, and multiple recurrences are frequent [3]. Thus, alternative therapeutic approaches to antibiotics are required. Among them, fecal microbiota transplantation and immunotherapy are promising. To prevent recurrences and trigger a long term protection against CDI, vaccination appears to be a rational strategy [4]. The toxins TcdA and TcdB are the main virulence factors responsible for the clinical signs. Several vaccines targeting these toxins are under investigation in clinical trials and seem to show efficacy after parenteral immunization (clinical trials Sanofi Pasteur NCT01887912, Pfizer NCT03090191). However, intestinal colonization is the first step of the *C*. *difficile* pathogenic process; therefore targeting colonization factors represents an interesting vaccine strategy. Indeed, immunity induced by this approach will reduce the colonization process of infection, limiting the first interaction between the bacterium and its host and therefore the faecal shedding of bacteria. Several studies have shown promising results and identified interesting vaccine candidates such as the Cwp84 protease and the flagellin FliC [5–7]. The precursor of the S-layer proteins (SlpA) has already been successfully tested [8–11]. Besides the identification of the antigenic target, a better knowledge of the protective immune response against *C*. *difficile* and the respective role of the local and systemic response will improve vaccine development [12]. Moreover, since CDI is strictly located in colonic mucosa, a mucosal immunization appears as a rational strategy to develop a local intestinal immunity. In addition, for vaccine design, the choice of the adjuvant related to the administration route is crucial to optimize immune response [13].

Cholera toxin (CT) remains the most commonly used adjuvant for mucosal immunization assays in animal models [14]. However, the use of CT is not possible in humans [15,16]. As alternative, several mucosal adjuvants are under investigations such as agonists of pattern recognition receptors (PRRs) capable of triggering the innate immune system. For instance, flagellins are known to be pathogen-associated molecular patterns (PAMPs) harboring a highly conserved domain involved in Toll-like receptor 5 (TLR5) interaction but also an antigenic domain variable among species [17–19]. Thus, bacterial flagellins play a crucial role in triggering innate and adaptive immunity at the systemic level and in mucosa associated lymphoid tissue (MALT) [20]. Few studies have investigated the adjuvant effect of flagellin [21]. For instance, *Salmonella* Typhimurium flagellin (FLA-ST), administered either via the intra-nasal or a parenteral route, has been demonstrated to have an effective adjuvanticity [22,23]. The interest in *C*. *difficile* flagellin FliC in vaccination has already been evaluated since FliC plays a key role in the pathogenesis ranging from bacterial colonization to gene regulation through immunomodulatory effects [24–28]. FliC immunogenicity has been proven by the detection of specific antibodies against FliC in CDI patients [29]. This immunogenicity was recently confirmed by parenteral immunization assays in mice and hamsters with FliC as antigen [6]. Recently, the interaction between *C*. *difficile* flagellin FliC and TLR5 was confirmed *in vitro* and *in vivo* with a similar activity compared to *Salmonella* Typhimurium flagellin (FLA-ST) [27,30,31]. These results suggest that FliC could also be used as an adjuvant in mucosal vaccination. An adjuvant able to stimulate the intestinal mucosal immune system and generate secretory Immunoglobulins A (sIgA) production could be essential in the development of an effective mucosal vaccine directed against enteropathogens such as *C*. *difficile*.

The main objective of this study was to characterize FliC as adjuvant. Firstly, we assessed the adjuvant property of *C*. *difficile* flagellin FliC in comparison to FLA-ST and CT using ovalbumin (OVA) as antigen in mice. We characterized the gut mucosal and systemic immune response according to adjuvants in immunized mice. Secondly, we confirmed the efficacy of FliC as an adjuvant when administered with the precursor of the S-layer proteins SlpA as antigen, in a mouse model of intra-rectal vaccination against *C*. *difficile* colonization.

## Materials and methods

### FliC and SlpA production

FliC and SlpA recombinant proteins were obtained and purified as previously described [9,27]. Briefly, the *slpA* and *fliC* genes (from the 630 and UK R20291 *C*. *difficile* strains, respectively) were cloned in frame with histidine tags. Then, plasmids were introduced by transformation into *E*. *coli* BL21. The SlpA and FliC proteins were obtained after expression by IPTG and purification by a single-step affinity chromatography using BD Talon cobalt affinity resin (BD Biosciences). FliC purification steps were verified by SDS-PAGE followed by Western blot.

### Animal models

Animal assays were performed with six weeks old female C57BL/6 J mice (Charles River’s laboratories France). For blood sampling and intra-rectal (IR) immunizations, mice were anaesthetized by intra-peritoneal (IP) injection of Ketamine 1000 (100mg/Kg), Rompun 2% (0.25mL/Kg).

Protocols involving animals and their care were conducted in conformity with the institutional guidelines that are in compliance with national and international laws and policies. All efforts were made to minimize animal suffering. Animals were humanely euthanized by cervical dislocation after anesthesia as approved by the Committee on the Ethics of Animal Experiments n°26 University of Paris-Sud and authorized by the French Ministery of Research (6164-201607l5154154l8).

### Vaccination regimen with ovalbumin as antigen

Ovalbumine (OVA) is a commonly used antigen in order to assess adjuvant activity [19,32]. In two distinct assays, a total of fourteen mice per group received intra-rectally (IR) 20μg of ovalbumin (ovalbumin grade VII, Sigma) with 7μg of adjuvant, either cholera toxin (CT) (List Biological Laboratories, INC), or FLA-ST (Standard flagellin from *S*. Typhimurium, Invivogen, France), or purified recombinant FliC. A control group of 14 mice received 20μg of ovalbumin in phosphate-buffered saline (PBS). In addition, to compare IR (mucosal) immunization with IP (parenteral) route of immunization with FliC, a group of eight mice received 20μg of ovalbumin with 7μg of purified recombinant FliC intraperitoneally (IP). Each group of mice received 3 identical doses: a first one on day 0, a second dose on day 15 and a last dose on day 30. At day 45, mice were humanely euthanized (guidelines of the "Animal Welfare Committee of the Université Paris Sud") for blood and intestinal content sampling and cell isolation from intestinal lamina propria.

### Evaluation of the immune response after immunization with different adjuvants co-administered with ovalbumin as antigen

#### Blood and intestinal content collection

Blood samples from all mice were withdrawn under anesthesia by cardiac puncture before each immunization on days 0, 15, and 30 and before their sacrifice on day 45, fifteen days after the last immunization. At this time, caecum and colon of each mouse were removed, opened and washed with 1mL of PBS containing a protease inhibitor cocktail (cOmplete^™^ Mini, Roche, France). Intestinal contents were treated as already described [10]; briefly they were clarified by a first centrifugation at 4,000×g for 10 min at (at 4°C), then the supernatant was centrifuged for 10 min at 12,000×g (at 4°C). The supernatants were collected and stored at −80°C until analysis.

#### Intestinal lamina propria cell isolation

Protocol was adapted from the method described by Weigmann *et al*.[33]. Briefly, caecum and colon from 5 immunized mice of each group were removed and washed at D45, when mice were sacrificed. Tissues were cut in small pieces and incubated under shaking at 37°C for 15 min in a pre-digestion medium and tissues were retained on a 100μm nylon cell strainer, this pre-digestion was repeated three times (PBS 1X, HEPES 10mM, EDTA 5mM). Tissues were then digested for 25 min on shaker at 37°C in digestion medium (20ml RPMI, HEPES 10mM, collagenase type IV 0.25 mg/ml). Digested tissues were disaggregated and filtered through a 70μm nylon cell strainer. Cells were washed with cell culture medium (DMEM, fetal calf serum 10%, penicillin 100U/ml, streptomycin 5μg/ml), and cell suspensions were centrifuged 20 min at 400×g at 4°C and resuspended with 1ml of cell culture medium. Living cells were counted using trypan blue exclusion test.

#### Detection of specific IgA and IgG by ELISA

For each mouse, OVA specific antibody levels in sera and intestinal samples were assessed using ELISA. Wells of a 96-well microtitre plate (MaxiSorp, Nunc) were coated with 10μg of OVA in carbonate/bicarbonate buffer pH 9.5. Microplates were washed with PBS/0.01% Tween 20 and then blocked with PBS/1% BSA (Bovine serum albumin) overnight at 4°C. A 100 μl aliquot of samples (dilution 1:20 for sera and 1:10 for intestinal contents) was added in each well and microplates were incubated for 30 min at 37°C. After washing, 100 μl of biotinylated anti-mouse IgG (dilution 1:20,000) or IgA (dilution 1:10,000) antibodies (Sigma) were added in each well and plates were incubated 30 min at 37°C. Then, after washing, 100 μl of HRP-conjugated streptavidin were added in each well (dilution 1:10,000 Thermo Scientific) and plates were incubated 30 min at 37°C, and finally, 100 μl of 3,3′,5,5′- tetramethylbenzidine (Sigma) were added in each well as development reagents. The reaction was stopped by addition of 100 μl of H2SO4 (1N) and the Optical Density (OD) at 450 nm was determined.

All samples were tested in duplicate and treated simultaneously to avoid inter-assay variations. Assays with antigen in the absence of sera served as negative controls and an OD two fold greater than the negative control defines the cut-off value [34]. Thus, mice with samples yielding an OD greater than the cut-off value were reported as positively responding to the immunization.

#### Detection of antigen-specific antibody producing cells by ELISPOT

MultiScreen-HA sterile plates (0.45 μm surfactant-free mixed cellulose ester membrane, Millipore) were coated over night at 4°C with 50 μl of OVA (5 μg/ml) in sterile PBS pH 7.5. After two washes with ultra pure water, wells were blocked with 150μl of cell culture medium for 2h at 37°C. A 100 μl aliquot, containing 50,000 isolated living cells were added per well in quadruplate and plates were incubated over night in a cell incubator (5% CO_2_, 37°C). Plates were washed three times with washing buffer (PBS, Tween20 0.01%) and twice with PBS only. Biotin-conjugated anti mouse IgG or IgA was added at a dilution of 1:2000 and plates were incubated for 2h at room temperature. After washing, streptavidin-HRP (Thermo scientific) was added and plates were incubated 30 min at 37°C. After washing, reactions were detected with 3-amino-9-ethyl-carbazole (AEC, Sigma) in acetate buffer according to manufacturer’s instructions. After 20 min, reaction was stopped by washing abundantly with water for 2 min [35]. Plates were read using an AID ELISPOT reader. Results were expressed in Spot Forming Units (SFU) per 10^6^ cells.

### Intra-rectal vaccination regimen with SlpA as antigen

One group of 6 mice received intra-rectally 50μg of purified recombinant SlpA and 5μg of cholera toxin. Another group of 6 mice received intra-rectally 50μg of purified recombinant SlpA and 5μg of FliC as adjuvant. The control group of 6 mice received intra-rectally 80μl of PBS. Mice were vaccinated on days 0, 15, 30. At day 45, mice received an antibiotic treatment in drinking water for seven days. The cocktail of antibiotics was composed of kanamycin (0.4mg/mL), gentamicin (0.035mg/mL), colistin (850U/mL), metronidazole (0.215mg/mL), and vancomycin (0.045 mg/mL) [36]. The concentration of the antimicrobial mixture was calculated based on the average weight of mice and their expected water consumption. On the third day of treatment (day 48), a dose of clindamycin (10mg/Kg) was IP-administered. One day after the end of antibiotic treatment (day 53), mice were challenged by oral administration of 8.10^2^ *C*. *difficile* spores of the 630 strain [37]. Vaccination trials were performed twice.

Spores were prepared as previously described [38]. Briefly, cultures of the 630 toxigenic strain of *C*. *difficile* were grown anaerobically at 36°C for 5–7 days, on blood/agar plates. The cultures were harvested into 10 mL of PBS, were washed in PBS, and were then heat-shocked at 56°C for 10 min. The spores were centrifuged, resuspended in sterile water and conserved at 4°C until use. Spores were quantified by culture of 10-fold serial dilutions on Columbia agar plates supplemented with 4% horse blood and sodium taurocholate (0.1%).

#### *C*. *difficile* detection in mouse fecal samples

To assess the intestinal colonization rate of *C*. *difficile*, fecal pellets from each mouse were cultured before antibiotic administration to ensure the absence of *C*. *difficile* before challenge and daily after *C*. *difficile* challenge for ten days. Fecal samples were processed as previously described [10]. Briefly, a fecal suspension of 10 mg/mL in PBS was prepared, and 100μL of ten-fold serial dilutions were cultured in anaerobiosis on Columbia agar containing 5% of horse blood, 25% (w/v) of D-cycloserine, and 0.8% (w/v) of cefoxitin and sodium taurocholate (0.1%). Typical fluorescent colonies were counted under UV light (312 nm). By this method, the threshold of *C*. *difficile* detection in animal feces was 10 CFU/mg.

### Statistical analysis

Normality was verified using Kolmogorv-Smirnov test and Shapiro-Wilk test. For normally distributed data, an unpaired student t-test was used. For non-normally distributed data Mann-Whitney U-test was used. A p-value *p* < 0.05 was considered to indicate statistical significance.

## Results

### Evaluation of the gut mucosal immune response induced by FliC, FLA-ST, CT as adjuvant and OVA as antigen

To compare the gut mucosal adjuvant potency of FliC with already known adjuvants FLA-ST and CT, we immunized mice with OVA via IR (mucosal) or IP (parenteral) route. Then, we evaluated and compared the induction of IgA and IgG specific to OVA in the intestinal contents and sera. In addition, we detected IgA and IgG producing cells in the lamina propria from mouse colon and ceacum after immunizations.

**Fig 1:**
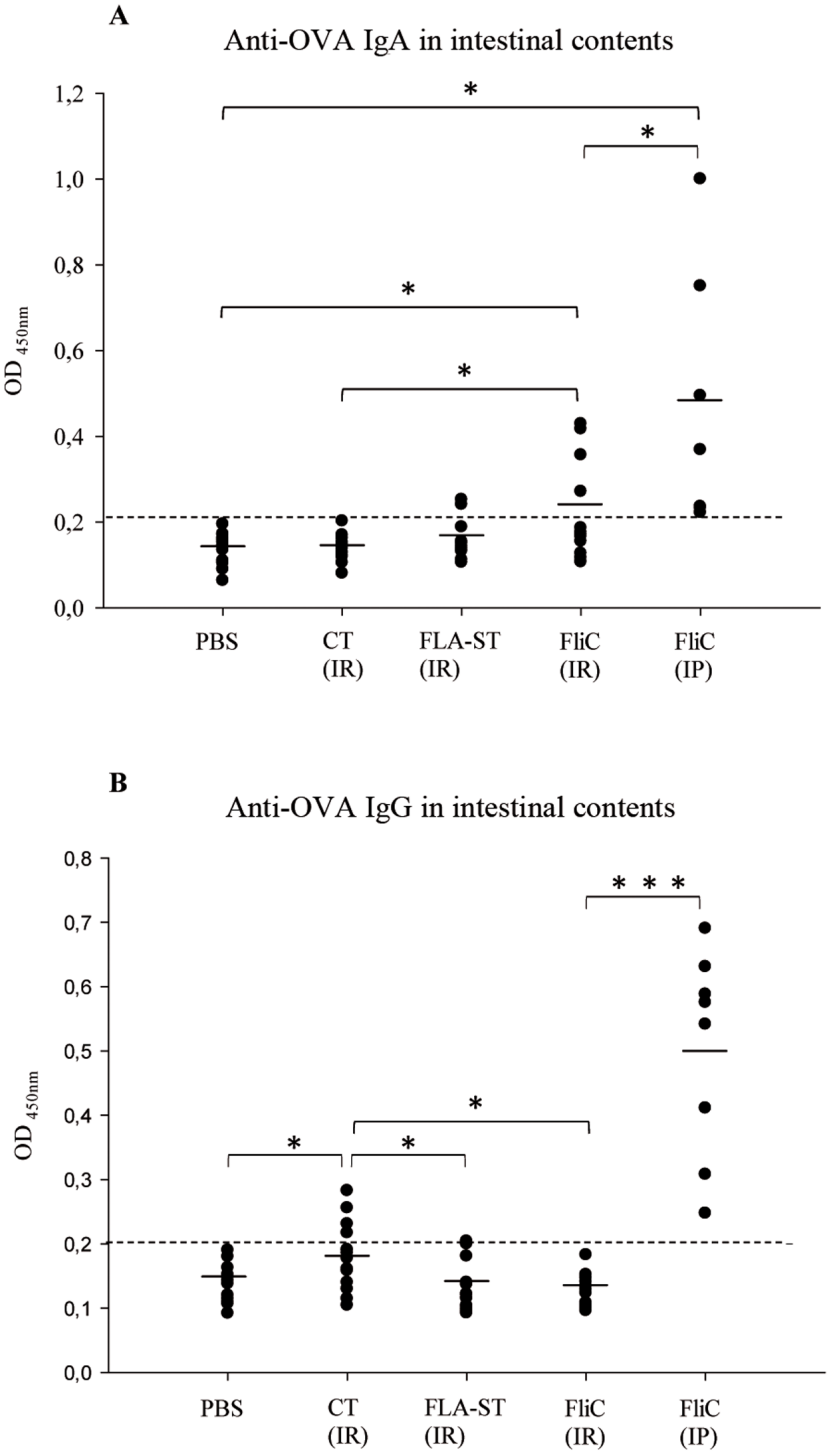
Anti-OVA antibody levels in intestinal contents of immunized mice. Immunization by intra-rectal route with ovalbumin as antigen (OVA), with cholera toxin (CT) (n = 14), *Salmonella* Typhimurium flagellin (FLA-ST) (n = 14), *C*. *difficile* flagellin (FliC) (n = 14) respectively as adjuvant and PBS administration for the control group (n = 14). Immunization by intra-peritoneal route with OVA as antigen and FliC as adjuvant (FliC IP) (n = 8). (A) Anti-OVA IgA level; (B) anti-OVA IgG level. *: statistically significant difference *: 0.01<*p*<0.05; **: 0.001<*p*<0.01; ***: *p*<0.001 (student t-test). Dotted-line corresponds to the cut-off value.

In intestinal contents of IR-immunized mice with FliC as adjuvant, 4 out of 14 mice showed an anti-OVA IgA response higher than the defined cut-off value (Fig 1A). Despite inter-individual variation, a significant greater production of OVA specific IgA was observed with FliC as adjuvant compared to mice immunized with CT (*p* = 0.019) and PBS control mice (*p* = 0.020). However, the OVA specific IgA level remains low for these groups. Despite inter-individual variation, all IP-immunized mice with FliC as adjuvant presented an anti-OVA IgA response higher than the cut-off value. IP-immunized mice produced significantly more OVA specific IgA than IR-immunized mice with FliC (*p* = 0.013), FLA-ST (*p* = 0.033) or CT (*p* = 0.025) and PBS control mice (*p* = 0.038).

Regarding IgG in intestinal contents, no IR-immunized mice with FliC or FLA-ST as adjuvant displayed OVA specific IgG in the intestinal lumen higher than the cut-off value. However, for IR-immunized mice with CT, only 4 out of 14 mice presented an anti-OVA IgG response higher than the cut-off value and with a significant difference compared to IR-immunized mice with FliC, FLA-ST and PBS control mice (*p* = 0.016). Of note, IP-immunized mice with FliC presented significantly more anti-OVA IgG in intestinal contents than IR-immunized mice whatever the adjuvant used (*p* < 0.001) (Fig 1B).

To further complete the characterization of the induced local immune response, we also detected antibody producing cells specific to OVA in the intestinal lamina propria.

**Fig 2:**
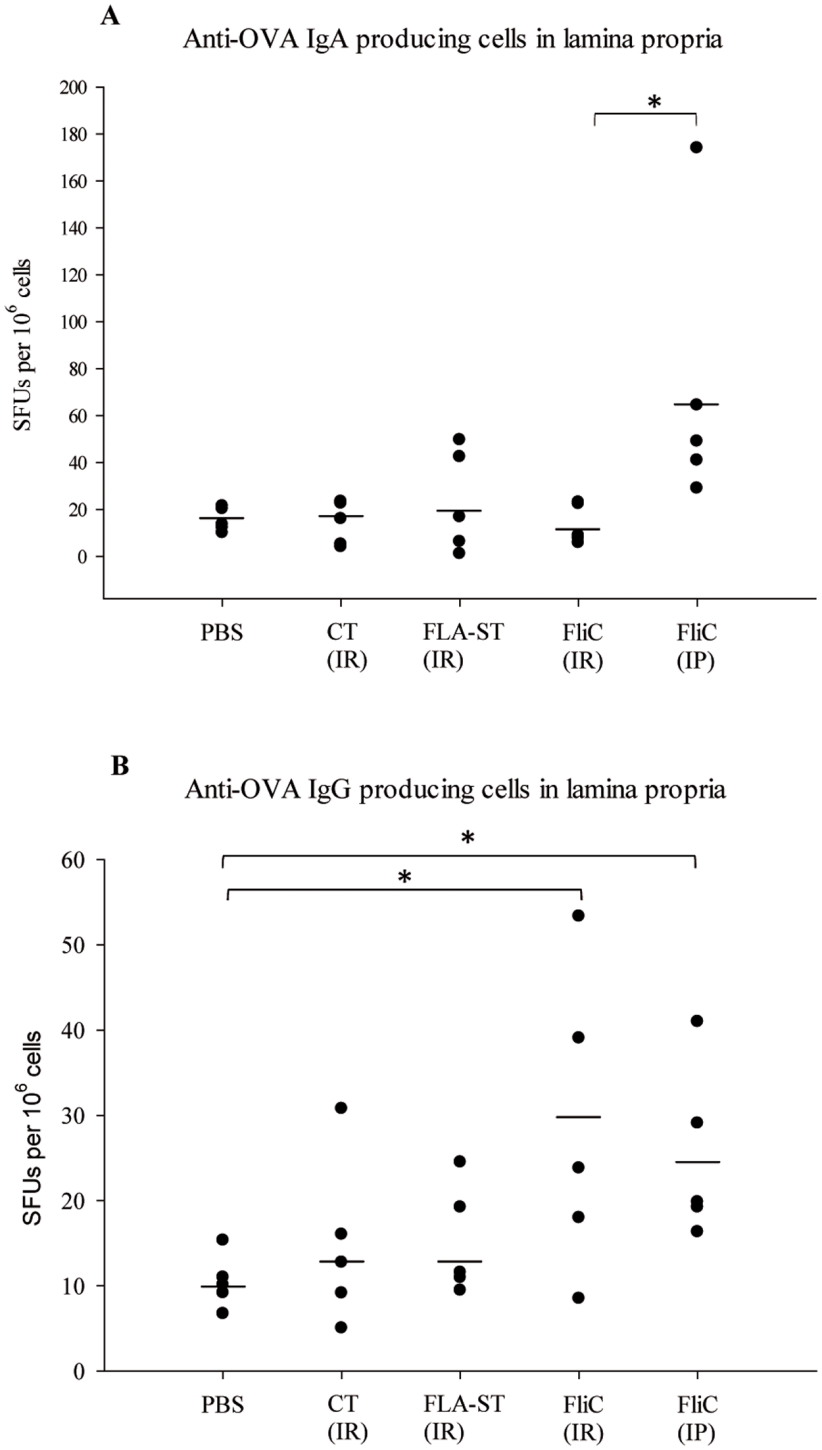
Number of antibody producing cells specific to OVA in the intestinal lamina propria of immunized mice expressed in spot forming units (SFUs). Immunization by intra-rectal route with ovalbumin as antigen (OVA), with cholera toxin (CT) (n = 5), *Salmonella* Typhimurium flagellin (FLA-ST) (n = 5), *C*. *difficile* flagellin (FliC) (n = 5) respectively as adjuvant and PBS administration for the control group (n = 5). Immunization by intra-peritoneal route with OVA as antigen and FliC as adjuvant (FliC IP) (n = 5). (A) Anti-OVA IgA producing cells count; (B) anti-OVA IgG producing cells count *: statistically significant difference *p* < 0.05 (Mann-Whitney U-test).

We looked at anti-OVA IgA producing cells in the intestinal lamina propria: for IR-immunized mice, no significant difference compared to the PBS control was observed independently of the adjuvant. In contrast, IP-immunized mice with FliC displayed significantly more anti-OVA IgA producing cells than IR-immunized mice (*p* = 0.040) (Fig 2A).

Concerning anti-OVA IgG producing cells in the intestinal lamina propria, despite important inter-individual variation, significantly more cells were detected in IR- and IP-immunized mice with FliC as adjuvant compared to PBS control (*p* = 0.041, *p* = 0.014 respectively) (Fig 2B). No significant difference was observed between mice IR-immunized with CT or FLA-ST and PBS control.

### Evaluation of the systemic immune response induced by FliC, FLA-ST, CT as adjuvant and OVA as antigen

**Fig 3:**
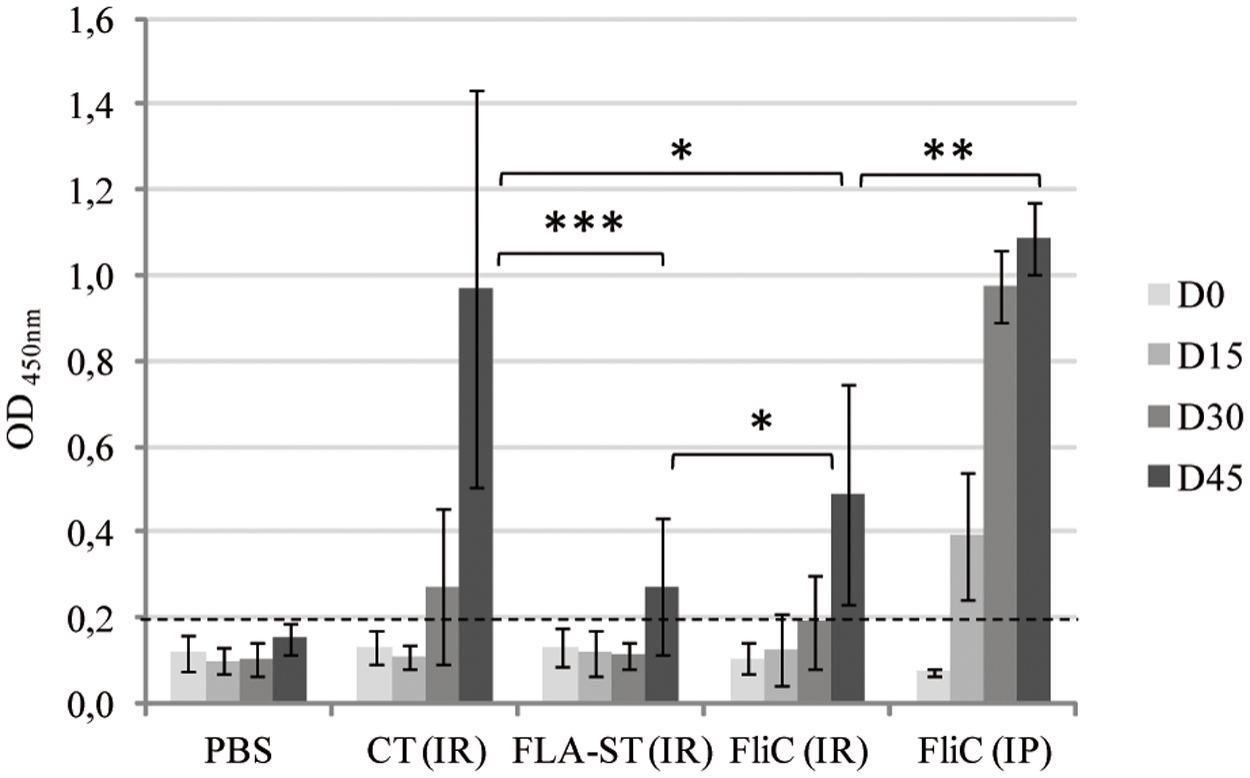
Anti-OVA IgG level in sera of immunized mice. Immunization by intra-rectal route with ovalbumin as antigen (OVA), with cholera toxin (CT) (n = 14), *Salmonella* Typhimurium flagellin (FLA-ST) (n = 14), *C*. *difficile* flagellin (FliC) (n = 14) respectively as adjuvant and PBS administration for the control group (n = 14). Immunization by intra-peritoneal route with OVA as antigen and FliC as adjuvant (FliC IP) (n = 8). D0: before immunization, D15: first immunization, D30: first boost, D45: second boost. *: statistically significant difference *: 0.01<*p* < 0.05; **: 0.001<*p*<0.01; ***: *p*<0.001 (student t-test). Dotted-line corresponds to the cut-off value.

At the systemic level, the three adjuvants tested were able to induce an OVA specific IgG response compared to the PBS control group (Fig 3). However, three immunizations were needed via the IR route, whereas only two were sufficient via the IP route with FliC as adjuvant. The intensity of the induced response was different according to adjuvant. At day 45, IR-immunized mice with CT as adjuvant presented a significant greater level of OVA specific IgG than IR-immunized mice with either FLA-ST or FliC (*p* < 0.001). Interestingly, FliC IR-immunization induced a significantly higher level of OVA-specific IgG than FLA-ST (*p* = 0.048). However, the highest level of OVA-specific IgG in sera was observed in IP-immunized mice with FliC as adjuvant, with a significant difference compared to IR-immunized with FliC as adjuvant (*p* = 0.0069).

### Intra-rectal vaccination against *C*. *difficile* colonization using FliC as adjuvant and SlpA as antigen

**Fig 4:**
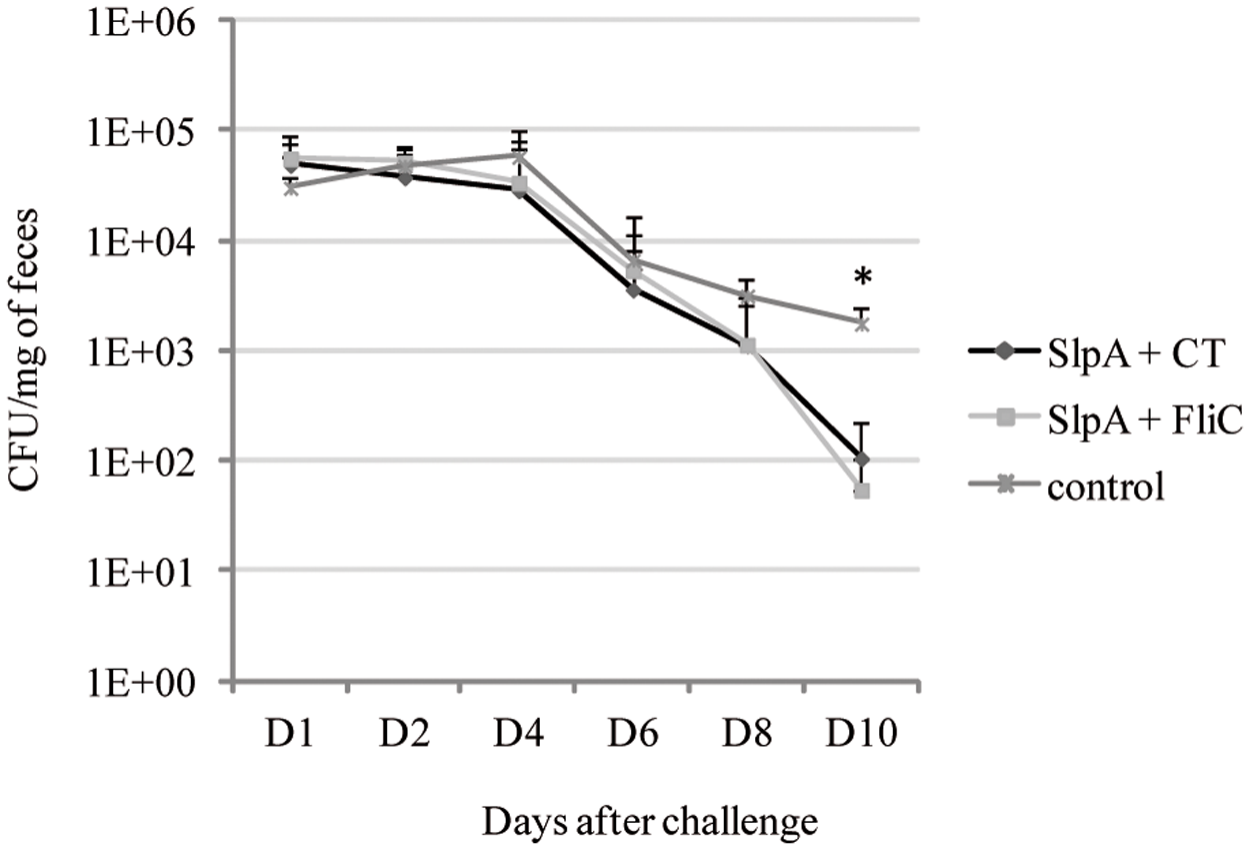
*C*. *difficile* colonization in vaccinated mice after challenge. *C*. *difficile* count from day 1 to day 10 in feces of mice intra-rectally vaccinated with SlpA as antigen and FliC (n = 12) or CT (n = 12) as adjuvant compared to control group (n = 12). *: statistically significant difference between the control and vaccinated groups *p* < 0.05 (Mann-Whitney U-test).

In order to develop a mucosal vaccine to prevent *C*. *difficile* intestinal colonization, we tested the ability of FliC as mucosal adjuvant co-administrated with SlpA as antigen in a mouse model of infection against *C*. *difficile* colonization. Mice were IR-vaccinated with SlpA either with CT or FliC as adjuvant. The control group received only PBS. Then, mice were challenged with the virulent *C*. *difficile* strain 630. As shown in Fig 4, from day 4 after challenge to day 10, the *C*. *difficile* fecal bacterial count was always lower in the vaccinated groups than in the control group. At day 10, the difference between vaccinated mice and non-vaccinated mice was statistically significant (*p* < 0,002).

## Discussion

In this study, we assessed the adjuvant property of *C*. *difficile* flagellin FliC in comparison to flagellin FLA-ST and CT, using OVA as antigen in mice. Then, we tested *C*. *difficile* flagellin FliC as adjuvant as an alternative to CT in IR vaccination strategy against *C*. *difficile*.

Many studies have described the adjuvant property of flagellin inducing both mucosal and systemic immunity in the context of a broad range of recombinant vaccines administered via the intra-nasal route or a parenteral route in animal models [23]. Strindelius *et al*. showed that immunization with the flagellin of *Salmonella enterica* serovar Enteritidis alone via the oral route was able to induce a systemic IgM-IgG response and a specific mucosal IgA response in the intestine [21]. Recently, Ghose *et al*. approached the question of *C*. *difficile* flagellin adjuvanticity in animal models via a parenteral route using an antigenic cocktail composed of receptor binding domains of TcdA and TcdB toxins [6]. They observed a significant increase of anti-TcdA specific IgG in sera of the FliC-adjuvanted mouse group compared to the non adjuvanted group. Of note, no difference in anti-TcdB IgG production was observed. For these authors, TcdA as vaccine candidate could also partially acts as an adjuvant. To our knowledge, the adjuvanticity of *C*. *difficile* flagellin FliC has not yet been studied via mucosal immunization.

In this study, we assessed mucosal and systemic FliC adjuvanticity after IR- and IP-immunization by evaluating the gut mucosal OVA specific IgA and IgG response and in parallel the systemic OVA specific IgG response. We showed that *C*. *difficile* flagellin FliC, used as adjuvant, was able to promote a gut mucosal and systemic OVA specific antibody response.

According to the route of immunization, FliC as adjuvant can induce a local antigen (Ag) specific IgA response in intestinal mucosa at different levels and with important inter-individual variations. Only a few IR-immunized mice with FliC showed a weak positive Ag-specific IgA response (4/14, 29%). The number of Ag-specific IgA producing cells in the intestinal lamina propria was significantly greater for IP-immunized mice than for IR-immunized mice. This was correlated with a significant higher Ag-specific IgA level in intestinal content of IP-immunized mice with FliC than IR-immunized mice with FliC and control mice. Taken together, these results showed that IP-immunization with FliC allows a full Ag-specific IgA response.

Flores-Langarica *et al*. showed that IP-immunization with FLA-ST resulted in a pronounced IgA switching of FLA-ST-specific B cells in mesenteric lymph nodes four days after boost [39]. In comparison, in our study, IgA antibody producing cells were isolated in the intestinal lamina propria 15 days after a second boost.

This local IgA response was associated with a local Ag-specific IgG response. All IP-immunized mice with FliC developed a gut mucosal Ag-specific IgG response as attested by anti-OVA IgG level in intestinal contents and the presence of anti-OVA IgG producing cells in the intestinal lamina propria. Surprisingly, Ag-specific IgG were detected only in intestinal content of IP-immunized mice although Ag-specific IgG producing cells in lamina propria were present after IP and IR immunization with FliC.

Independently of the route of administration, the local antibody response induced by FliC as adjuvant was associated with a systemic response as attested by the presence of Ag-specific IgG in immunized mouse sera. As expected, this IgG response was greater with IP-immunization compared to IR-immunization. Of note, IR-immunized mice with FliC presented significantly more circulating Ag-specific IgG compared to mice immunized with FLA-ST.

It should be noted that the detection of immunoglobulins by ELISA reflects a global production whereas antibody producing cell detection by ELISPOT corresponds to a cell number at a specific time point. However, the discrepancy between the number of Ag-specific antibody producing cells in intestinal lamina propria and the antibody level in intestinal content may suggest that IR and IP immunizations induce different immune responses and at different levels. For instance, Fougeron *et al*. showed that the intranasal adjuvant activity of flagellin is linked to the indirect activation of lung dendritic cells but not via TLR5-mediated signaling from the epithelial cells [22]. In contrast, Kinnebrew *et al*. showed that flagellin immunization by parenteral route directly stimulates TLR5 signaling in lamina propria dendritic cells [40]. The presence of Ag-specific IgG in the intestinal contents could be explained by a high systemic induced response and the passive diffusion or FcRn-mediated transport of IgG through the epithelium [41]. Here, IR immunization with FliC induced a low IgG response locally despite the presence of Ag-specific IgG producing cells. This is in accordance with the low level of IgG in sera and the need of three immunizations to induce a significant IgG response. In contrast, IP immunization can induce a strong systemic IgG response, attested by the presence of Ag-specific IgG producing cells in intestinal lamina propria, the high level of Ag-specific IgG in sera and the need of only two immunizations to induce an IgG response.

The slight differences of immune response induced by FliC and FLA-ST after IR-immunization could be explained by their origin. Although TLR5 binding domain is highly conserved, the hypervariable domains of FLA-ST and FliC are different. Nempont *et al*. showed that the hypervariable domain of flagellin is essential to trigger systemic innate immunity but not to stimulate the mucosal innate immunity and adjuvanticity to foreign antigens, suggesting distinct mechanisms of induction in systemic and mucosal compartments [19].

Adjuvant capacity of CT and flagellins derives from different mechanisms. Indeed CT is not a TLR-agonist, and is internalized through the glycosphingolipid GM1-ganglioside receptor. CT has been widely experimentally used as mucosal adjuvant in animals and several studies have investigated the adjuvanticity of CT in many different vaccination regimens and animal models. After oral immunization, CT has been shown to: i) increase permeability of the intestinal epithelium leading to enhance uptake of co-administered antigen thus facilitating antigen presentation by a variety of cell types; ii) promote isotype differentiation in B cells leading to increased IgA production; iii) induce complex stimulation as well as inhibition effects on T-cell proliferation and cytokine production [42–44]. In our study, after IR-immunization with CT, we observed a higher IgG systemic response than those obtained in FliC, FLA-ST and PBS control groups. Surprisingly, this systemic response was not associated with a local IgA response. In particular, we observed a greater level of Ag-specific IgA in intestinal content after immunization with FliC than CT as adjuvant.

In order to develop a vaccine against *C*. *difficile* administered by mucosal route, CT has proved efficacy as adjuvant in IR vaccination assays against *C*. *difficile* [5,9–11]. However, bacterial flagellin represents a potential good alternative as adjuvant since it seems to be safe and effective administered via a mucosal route and at very low dose as attested in mouse [21] and in non-human primate models [45,46]. In addition, it has been reported that flagellin seems to be effective as adjuvant in the aged mouse immune system, which is promising for a vaccine targeting the elderly such as *C*. *difficile* vaccine.

As FliC seems to be a promising adjuvant, we tested it as an alternative to CT in mucosal vaccination strategy against *C*. *difficile*. Thus, we compared FliC and CT as adjuvant IR-administered with a surface protein of *C*. *difficile* (SlpA) involved in gut colonization as antigen [9]. Immunized mice were less colonized than non-immunized mice with a significant difference at day 10. Despite a different systemic antibody response between the two adjuvanted groups (significant higher level of seric SlpA specific IgG in CT group compared to FliC group; S1 Fig), the two groups of mice displayed the same kinetics of gut colonization by *C*. *difficile*. This suggests the potential role of gut mucosal IgA response in addition to systemic IgG response in FliC immunized mice. In this study, CT and FliC showed the same efficacy. These results suggest that *C*. *difficile* flagellin FliC could be used as adjuvant in mucosal vaccine strategy against CDI targeting colonization factors as an alternative to CT, which has no perspective of use in human vaccination.

## Supporting information

S1 Fig Anti-SlpA IgG level in sera of immunized mice. Immunization by intra-rectal route with SlpA as antigen and with cholera toxin (CT) (n = 12) or C. difficile flagellin (FliC) (n = 12) as adjuvant. D0: before immunization, D45: after the last immunization. * statistically significant difference p<0.05 (Mann-Whitney U-test).
(TIF)
