# Supplementary figures and images for: Clostridium difficile flagellin FliC: Evaluation as adjuvant and use in a mucosal vaccine against Clostridium difficile
Source: PLoS One. 2017 Nov 27;12(11):e0187212. doi: 10.1371/journal.pone.0187212 (PMC5703446; doi:10.1371/journal.pone.0187212)

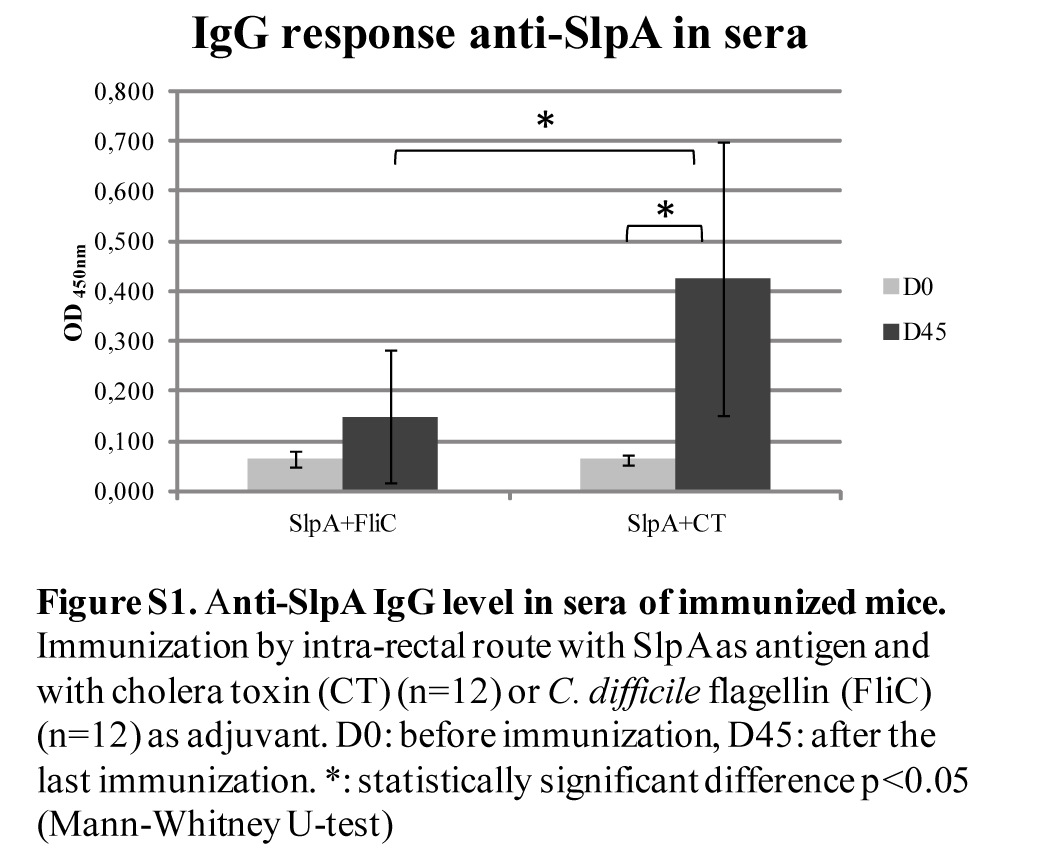

Supplement: S1 Fig — Immunization by intra-rectal route with SlpA as antigen and with cholera toxin (CT) (n = 12) or C. difficile flagellin (FliC) (n = 12) as adjuvant. D0: before immunization, D45: after the last immunization. * statistically significant difference p<0.05 (Mann-Whitney U-test). (TIF) [file pone.0187212.s001.tif]
